# Simple and Scalable Chemical Surface Patterning via Direct Deposition from Immobilized Plasma Filaments in a Dielectric Barrier Discharge

**DOI:** 10.1002/advs.202200237

**Published:** 2022-03-27

**Authors:** Annaëlle Demaude, Kitty Baert, David Petitjean, Juliette Zveny, Erik Goormaghtigh, Tom Hauffman, Michael J. Gordon, François Reniers

**Affiliations:** ^1^ Faculty of Sciences Chemistry of Surfaces Interfaces and Nanomaterials (ChemSIN) Université libre de Bruxelles Avenue F.D. Roosevelt 50, CP 255 Brussels B‐1050 Belgium; ^2^ Faculty of Engineering Department of Materials and Chemistry Electrochemical and Surface Engineering Research Group (SURF) Vrije Universiteit Brussel Pleinlaan 2 Brussels B‐1050 Belgium; ^3^ Structure and Function of Biological Membranes Center for Structural Biology and Bioinformatics Université libre de Bruxelles Avenue F.D. Roosevelt 50, CP 206/2 Brussels B‐1050 Belgium; ^4^ Department of Chemical Engineering Eng II #3351 University of California – Santa Barbara Santa Barbara CA 93106‐5080 USA

**Keywords:** atmospheric plasmas, patterned surfaces, streamers, X‐ray photoelectron spectroscopy

## Abstract

In this work, immobilization of the often unwanted filaments in dielectric barrier discharges (DBD) is achieved and used for one‐step deposition of patterned coatings. By texturing one of the dielectric surfaces, a discharge containing stationary plasma filaments is ignited in a mix of argon and propargyl methacrylate (PMA) in a reactor operating at atmospheric pressure. From PMA, hydrophobic and hydrophilic chemical and topographical contrasts at sub‐millimeter scale are obtained on silicon and glass substrates. Chemical and physical characterizations of the samples are performed by micrometer‐scale X‐ray photoelectron spectroscopy and infrared imaging and by water contact angle and profilometry, respectively. From the latter and additional information from high‐speed imaging of the plasma phase and electrical measurements, it is suggested that filaments, denser in energetic species, lead to higher deposition rate with higher fragmentation of the precursor, while surface discharges igniting outwards the filaments are leading to smoother and slower deposition. This work opens a new route for a one‐step large‐area chemical and morphological patterning of surfaces at sub‐millimeter scales. Moreover, the possibility to separately deposit coatings from filaments and the surrounding plasma phase can be helpful to better understand the processes occurring during plasma polymerization in filamentary DBD.

## Graphical abstract

Sub‐mm patterned thin films of plasma polymerized propargyl methacrylate are obtained using a dielectric barrier discharge device. Plasma filaments are immobilized by texturing one dielectric with beads and lead to faster deposition than the surrounding plasma phase. Wettability contrasts were observed and related to differences in the chemical composition of the films by micro‐X‐ray photoelectron spectroscopy and micro‐infrared analysis.

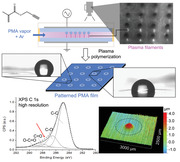

## 1 Introduction

Technological advances over the past decade have created an increasing need for complex and sophisticated materials. In particular, the local modification of surface chemistry and/or topography at sub‐millimeter scales has raised interest in a broad range of fields in fundamental research, as well as for practical applications. In biology, for example, nano/micropatterning of areas either supporting or inhibiting cell adhesion has been intensively used to study cell growth and interactions, as well as to control cell differentiation.^[^ ^1^, ^2^, ^3^, ^4^ ^]^ Controllable surface wettability patterns at different scales has been developed for many uses in microfluidics, water harvesting from humid air mimicking desert beetles, enhancing boiling heat transfer or liquid shaping and transport.^[^ ^5^, ^6^, ^7^, ^8^, ^9^ ^]^ Surfaces with chemical or topographical texture also find numerous applications in electronics and photonics.^[^ ^10^, ^11^, ^12^ ^]^ Although methods for surface patterning are many, there remain challenges in developing processes that are easy, cost‐effective, and scalable. Current techniques are generally based on selective layer deposition with the use of masks, lift‐off processes, or motif imprinting from mold to substrate, all of which often involve many steps.^[^ ^13^, ^14^, ^15^, ^16^ ^]^ Direct deposition/surface modification with energy beams is also common, but can be time‐consuming as they generally require low pressure environments and/or can only treat small areas.^[^ ^17^, ^18^ ^]^ In this realm, plasma technologies have been the tools of choice because they allow thin‐film etching and deposition.^[^ ^19^, ^20^ ^]^ In particular, dielectric barrier discharges (DBD) at atmospheric pressure are an attractive choice for industrial scale surface processing because they are scalable to large areas and can be easily implemented on process lines without the need for complex vacuum systems. In a planar DBD, the plasma is generated by ionization of gas between two plate electrodes driven by an alternating current source with the presence of at least one dielectric barrier to avoid arc formation.^[^ ^21^ ^]^ At atmospheric pressure, DBDs generally appear filamentary, where short‐lived and micrometer‐scale microdischarges (streamers) randomly form between the electrodes. Nowadays, the characteristics of these filaments and their ignition mechanisms are quite well understood. Plasma filaments are denser in electrons and ions than the surrounding gas that gathers and transports long‐lived species and absorbs the energy dissipated in microdischarges.^[^ ^22^, ^23^, ^24^ ^]^ As such, deposition or surface modification by filaments will be different from the surrounding environment. For example, Polonskyi et al. used self‐organized streamers in a DBD to locally modify the wettability of PMMA layers.^[^ ^25^ ^]^ Jiang et al. observed higher deposition rates near filaments compared to other locations in a DBD during plasma polymerization of acetylene, resulting in inhomogeneous material growth due to the random distribution of streamers on the substrate surface.^[^ ^26^ ^]^ Bröcker et al. recently deposited a sub‐millimeter width HMDSO thin film “dot” using a single filament in a DBD with a pin‐to‐plate electrode arrangement to study ionic contributions to HMDSO film growth.^[^ ^27^ ^]^ However, because of their random nature and short lifetime (≈10–100s ns), the potential of plasma filaments as microreactors for localized deposition has, to our knowledge, not been exploited for localized and large‐scale surface deposition yet. In this work, a DBD with a textured dielectric was used to immobilize plasma filaments in a mixture of Ar and propargyl methacrylate (PMA, a reactive precursor for plasma polymerization) and led to deposition of a thin film with patterned morphologies and wettability contrasts. The method for surface patterning presented in this paper is quite straightforward and, given the existing literature on thin film deposition with DBDs, could potentially be used with any precursor. The morphology of patterned coatings was characterized by profilometry and found to correlate with high‐speed images of filaments in the discharge; spatial differences in film chemistry were investigated by water contact angle (WCA), micrometer‐scale X‐ray photoelectron spectroscopy (XPS), and micrometer‐scale infrared imaging.

## 2 Results and Discussion

The DBD reactor used in this work is made from transparent materials (glass dielectrics and stainless steel meshes as electrodes), which enables to see the plasma discharge by looking from the top of the reactor (see the Experimental Section for complete description). In addition, the position of the (un)textured top dielectric and the bottom dielectric can be exchanged to see different parts of the discharge or deposit films on a substrate.

**Figure 1:**
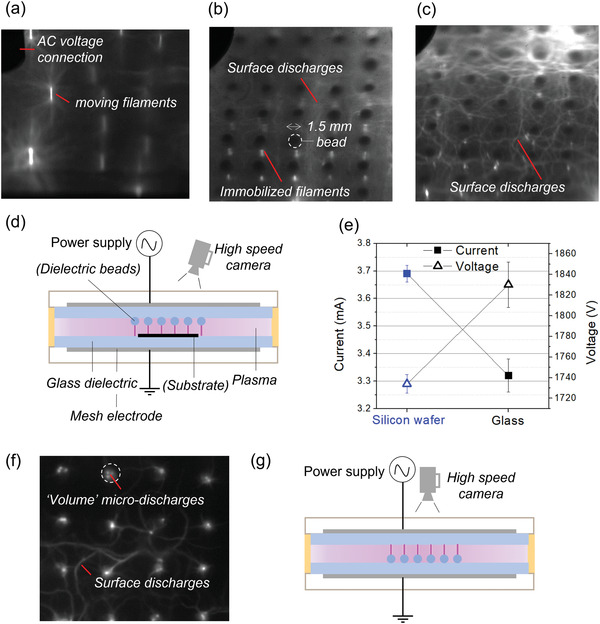
High‐speed images (1 ms exposure time) of streamer microdischarges generated at 10 W in a DBD plasma using a) an upper dielectric that was left untextured or b) an upper dielectric textured with ceramic beads without substrate and c) with a silicon wafer substrate placed on the bottom dielectric as schemed in (d). e) The arithmetic mean current and voltage ± standard deviations (two‐sided Student's test, *n* = 10, *P* = 8 × 10^−6^ for V and 2 × 10^−7 ^for I, *α* = 0.05) dissipated in a 6 W discharge with glass versus Si substrates. f) A single frame (0.1 ms exposure time) top view of a 10 W discharge with the textured dielectric on the bottom as schemed in (g). See the Experimental Section for more details on the DBD setup. Conditions: Ar flow = 2 L min^−1^, PMA flow = 0.03 L min^−1^, *f* = 24 680 Hz.

**Figure** **1** shows images of the DBD plasma discharge ignited in an argon/propargyl methacrylate (PMA) mix with untextured (a) and textured upper dielectrics (b) as schemed in (d). In the latter case, stationary plasma filaments are ignited preferentially under the beads (white dashed circles) used for texturing, whereas, for the untextured case, the filaments move randomly in between the dielectrics. Indeed, the presence of the beads locally reduces the discharge gap, which in turn, locally reduces the voltage required for gas breakdown and hence promotes the formation of microdischarges at specific locations. It should be noted that the bead material plays a role on the microdischarge formation as well, as does the nature of the dielectric.^[^ ^28^ ^]^ In addition to filaments, weaker discharges also radiate between the beads. These weaker filaments seem to increase when a silicon wafer is placed on the bottom dielectric under the beads (Figure 1c).

As the conductive nature of the substrate can locally change the electrical properties of the plasma, current and voltage measurements were performed using different substrates. Figure 1e shows that even though the dissipated power is the same in both cases, a lower mean voltage, and therefore greater current, was measured in the presence of silicon substrate than for the glass substrate (see voltage and current curves in the Supporting Information). This is most likely linked to the conductivity and secondary electron yield (*γ*) of the substrates. A more conductive substrate (Si) usually has greater *γ* and will tend to produce more electrons, leading to gas ionization at lower voltages compared to glass.^[^ ^29^ ^]^

The “tentacle‐like” phenomenon particularly visible on Si has been identified in other work as surface discharges (SD) induced by the accumulation of interface charges on the dielectric surface from the “volume” microdischarges (filament) initiated across the discharge gap. ^[^ ^30^ ^]^ The image in Figure 1f was recorded from the top of the reactor with the textured dielectric as the bottom‐side insulator as schemed in panel (g) at high frame/rate (10 000 fps ≈ 0.1 ms or 2.5 AC cycles exposure time) and shows that SD are initiated from the center of the beads. This image also shows that several microdischarges (bright dots) initiate over time at different places on a same bead (white circle), forming the filaments visible in Figure 1b,c and to the naked eye. The width of a microdischarge was ≈100 µm, much smaller than the diameter of the beads (≈1.5 mm) and agrees well with the theoretical size of a single microdischarge channel.^[^ ^22^ ^]^

**Figure 2:**
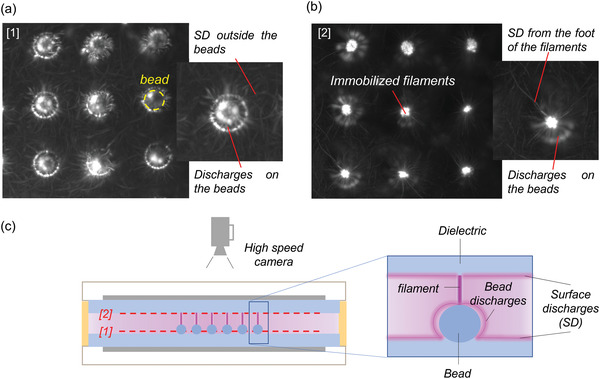
Overlay of 500 successive high‐speed frames (0.15 ms exposure time) of streamer microdischarges generated at 10 W in a DBD plasma with a textured dielectric placed on the bottom. The focal plane was set to the a) surface of the beads or b) on the surface of the upper dielectric, as schematized, respectively, by the [1] and [2] dashed lines in (c). Conditions: Ar flow = 2 L min^−1^, PMA flow = 0.03 L min^−1^, *f* = 24 680 Hz.

**Figure** **2** shows several successive high speed frames of the plasma discharge superimposed with the textured dielectric placed at the bottom of the reactor (like in Figure 1g). The focus was made on the top the beads (panel (a)) or on the inner surface of the top dielectric (panel (b)). The left overlay (panel (a)) suggests that microdischarges are also igniting between the top of the bead and the surface of the dielectric on which they are glued. These bead discharges are, themselves, also inducing SD ignition at the top of the dielectric as schematized in panel (c). In the right overlay (panel (b)), the microdischarges on the beads appear blurred and the SD on the top dielectric appear clearly. SD are thus generated on both dielectrics, but via different mechanisms, namely induced by the microdischarges ignited between the top of the beads and the dielectric upon which they are glued (bead discharges), or induced by the microdischarges ignited between the top of the beads and the opposing dielectric (filaments).

The deposition of PMA using untextured and textured dielectrics was next compared. The applied power was varied between 6 and 10 W and the coatings were deposited on glass and Si substrates. For the sake of brevity, the different patterned and (nonpatterned, NP) samples are named as follows: (“NP”—) “G” or “S” for glass or silicon wafer substrate, respectively—“6W” or “10W” depending on the applied power. Note that deposition is also possible with other beads spacings as shown in the Supporting Information.

**Figure 3. a):**
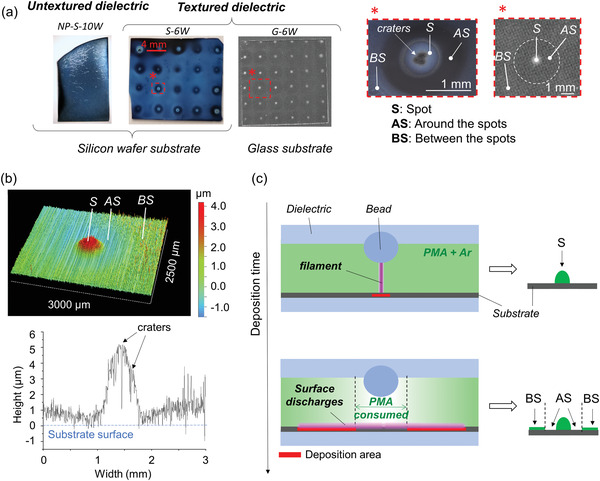
Pictures of PMA films deposited at 6 or 10 W on Si and glass substrate using an untextured (NP‐S‐10W) or a textured dielectric (S‐6W and G‐6W) and zoom on the different areas of interest (spot, “S”, around the spots, “AS” and between the spots, “BS”) of the patterned samples. b) A topographic mapping and profile of the “S”, “AS” and “BS” areas of a PMA patterned film deposited at 10 W on Si. c) Schematic of the deposition of the patterned films from the filaments and surface discharges, respectively. Conditions: Ar flow = 2 L min^−1^, PMA flow = 0.03 L min^−1^, *f* = 24 680 Hz, deposition time = 120 s.

**Figure** **3**a shows pictures of the NP‐S‐10W, S‐6W, and G‐6W samples. In all cases, the entire surface of the substrate was covered with a deposited film. However, the material deposition was markedly different, i.e., preferentially deposited into <1 mm diameter spots, in the case where the filaments were immobilized. As revealed by the closeup pictures of the S‐6W and G‐6W samples and by the topography mapping of the pattern (see Figure 3b), three different areas of interest can be defined: i) “spots” (S), appearing as circular hills, ii) a ring‐like depression “around a spot” (AS) with no or very thin and smooth film (few nm thick), and iii) “between spots” (BS). Additionally, there were small “craters” within the circular hills (i) for the Si sample only (see other profilometry profiles of spots from S‐10W and G‐6W samples in the Supporting Information).

The “S” and “BS” regions of the patterned samples are attributed to deposition from immobilized filaments and SD, respectively. The higher plasma density in microdischarges would lead to faster deposition than from SD, explaining the hill‐like shape of the spots. We hypothesize that the “AS” regions occur due to local consumption of the precursor inside the immobilized filaments and bead microdischarges (see Figure 3c). Indeed, as shown by Gao et al., SD occur only after the main filaments are formed and extinguished within a same voltage half‐cycle.^[^ ^30^ ^]^ As such, microdischarges would locally consume precursor in the gap, then, as the available precursor content increases again, i.e., at a certain distance from the filaments, the SD would lead to thicker film deposition.

Although the pattern is similar on both substrates, some differences are seen. First, the spots on Si have craters in their centers. The higher conductivity of Si could induce local heating and etching of the substrate where filaments are immobilized, and locally degrade the film. Second, “BS” regions of the S‐6W and S‐10W films are thicker compared to the corresponding regions on glass samples, which agrees with the greater number of surface discharges and higher current for Si (Figure 1d).

**Figure 4:**
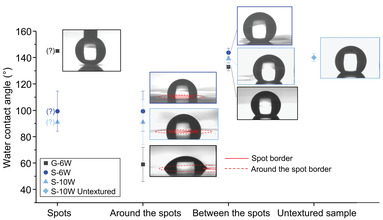
Water contact angle measurements of PMA films deposited at 6 or 10 W with or without immobilization of plasma filaments on Si (S‐6W, S‐10W, and S‐10W untextured) and glass (G‐6W) substrates (arithmetic means ± standard deviations over five measurements). Question marks indicate that the WCA could not be measured accurately, and only qualitative information on the wettability from images of the drop was obtained. Conditions: Ar flow = 2 L min^−1^, PMA flow = 0.03 L min^−1^, *f* = 24 680 Hz, deposition time = 120 s.

Prior work on atmospheric pressure DBD deposition with PMA resulted in “powdery” thin films with hydrophobic character and ≈140° contact angles.^[^ ^31^, ^32^ ^]^ To compare our results with these works, WCAs were measured for the G‐6W, S‐6W, S‐10W, and NP‐10W samples (see **Figure** **4**). In all cases, it appears that the “BS” areas and the untextured dielectric deposited films are hydrophobic. For the glass sample, the “AS” areas are hydrophilic, and the spots are very hydrophobic (a drop could not be deposited). For the S‐6W and S‐10W samples, the spots (“S” areas) did not repel water, and as the “AS” areas are small, these two regions were difficult to probe separately. Therefore, it could only be concluded that on Si, the “S” and “AS” areas are less hydrophobic than the “BS” areas. The similar wettability of the two Si samples suggests that the applied power has limited effect on the chemistry and/or roughness of the film in the tested range.

**Figure 5:**
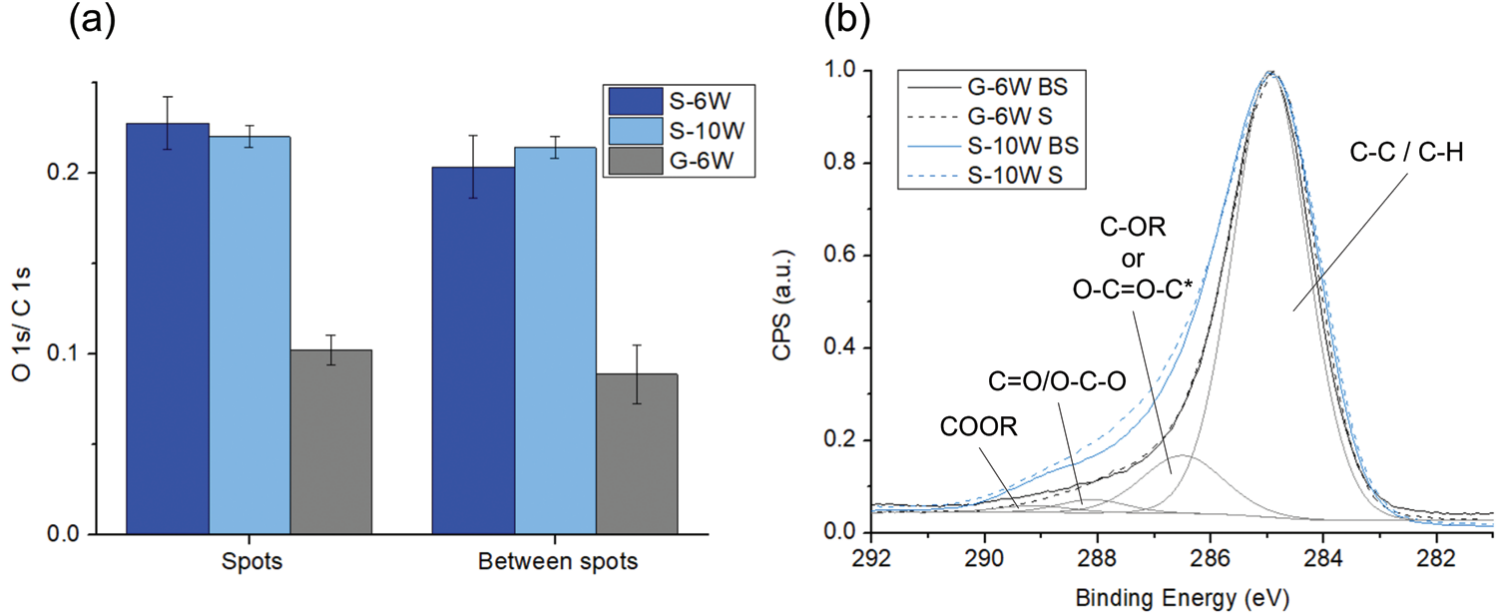
XPS analysis performed on the spots (“S”) and between the spots (“BS”) of PMA patterned films deposited at 6 and 10 W on glass or Si (G‐6W, S‐6W, and S‐10W). a) O 1s to C 1s peak intensity ratios (arithmetic means ± standard deviations, two‐sided Student's test, *n* = 3–8, *α* = 0.05, *P* = 0.01, 0.05, and 0.04 for G‐6W, S‐6W, and S‐10W, respectively). b) Average high‐resolution C 1s spectra (arithmetic means of at least three C 1s spectra acquired on the same analysis areas for the G‐6W and S‐10W samples, see the Supporting Information for spectra with the 95% confidence intervals) with detailed peak fitting (R = alkyl or H). Conditions: Ar flow = 2 L min^−1^, PMA flow = 0.03 L min^−1^, *f* = 24 680 Hz, deposition time = 120 s.

To investigate the effects of immobilized filaments and surface discharges on film chemistry, XPS analysis was performed on the spots and in‐between (“S” and “BS” areas) of the G‐6W, S‐6W, and S‐10W samples. **Figure** **5**a shows the O 1s‐to‐C 1s peak intensity ratio obtained from survey spectra; a slightly greater O/C ratio was noted for “S” area (*P*‐values close to 0.05) and the O/C ratio seems roughly constant throughout the different analyzed areas for all three samples (see survey spectra in the Supporting Information).

High‐resolution C 1s spectra for the S‐10W and G‐6W samples (Figure 5b) were also recorded on the same analysis areas and a fitting of the peak envelope was performed using four components: C—C/C—H, C—OR/O═C—O—C*, C═O/O—C—O, and COOR (with R═H or alkyl). The latter two peaks for the “S” and “BS” areas almost perfectly overlap for the G‐6W sample, while they deviate in oxygenated character for S‐10W (see the Supporting Information; the lower and upper 95% confidence intervals calculated for each region do not cross each other from 286.6 to 288.5 eV for S‐10W and overlap in the whole C 1s binding energy range for G‐6W). This means that chemical variations within the same deposited film are more important for S‐10W than for G‐6W, which agrees with the greater wettability contrast observed for the Si substrate.

Although all samples show the same hydrophobicity between the spots, but different wettability on the spots, the XPS measurements revealed lower oxygen content on both analyzed areas for G‐6W. This result can be counter‐intuitive, but the link between surface composition and wettability should not be compared between the two types of samples, as the nature of the substrate can influence the morphology of the film. Indeed, the latter properties also play a role on the wettability of plasma deposited films.

**Figure 6:**
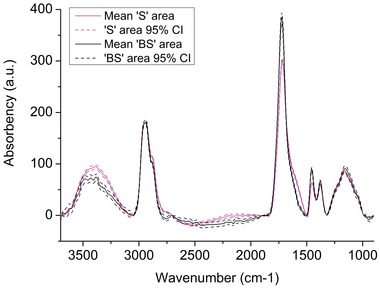
Arithmetic mean IR spectra (solid lines, normalized to the CH_2_/CH_3_ str. band) and 95% confidence intervals (*n* = 100, dashed lines) acquired on a patterned PMA (Al‐10W) film on the spots and in between (“S” and “BS” areas) deposited on aluminum. Conditions: Ar flow = 2 L min^−1^, PMA flow = 0.03 L min^−1^, *f* = 24 680 Hz, deposition time = 120 s.

Chemical variations in the S‐10W film suggested by the C 1s XPS spectra were supported by infrared analysis (IR‐ATR microscopy). For this analysis, a patterned PMA film was deposited on an aluminum substrate at 10 W at the same plasma conditions (Al‐10W sample). Spectra were selected on the “S” and “BS” areas from the IR image (see IR images at different wavenumbers in the Supporting Information). Their arithmetic means with the upper and lower 95% confidence intervals are shown in **Figure** **6**. The appearance of the aluminum sample was similar to a silicon wafer sample, with craters in the center of the spots. As aluminum is also a conductive substrate, the IR analysis of the Al‐10W sample was correlated with the XPS analysis of the S‐10W sample.

In both analysis areas, the spectra show the same vibrational bands, although with different intensities. The latter are assignable to OH (3500–2900 cm^−1^), CH_2_/CH_3_ (2900–2600 cm^−1^), and C═O (1850–1600 cm^−1^) stretches, CH bending (1500–1350 cm^−1^), and C–O stretches (1350–950 cm^−1^), which constitute a typical fingerprint of polyester. All the spectra were normalized to the CH_2_/CH_3_ stretch band for an easier comparison. The OH band is significantly more intense on the spot, which suggests that hydroxyl groups may be grafted during deposition from fragments generated in the discharge. The C═O stretching band is less intense on the spot, although broadened toward lower wavenumber. The location of this band can differentiate between ester and carboxylic groups when it appears in the 1750–1735 cm^−1^ versus 1720–1680 cm^−1^ ranges, respectively. This might suggest higher fragmentation of the precursor in the immobilized filaments and subsequent loss of the ester group and/or creation of a carboxylic acid moiety from the latter. The grafting of OH and COOH groups in the filament region could thus explain the greater wettability of the spots, and the results are in good agreement with XPS of the S‐10W sample revealing an increase in the oxygenated C 1s components on the spots.

## 3 Conclusion

In conclusion, plasma filaments in a dielectric barrier discharge were immobilized by locally reducing the discharge gap via texturing of the dielectric surface. In a mixture of Ar and PMA, thin films exhibiting patterned morphology and wettability were deposited on glass and Si at different applied powers and compared with a PMA film obtained with no control over filament location. The pattern in the deposited film is likely associated with the higher energy density inside plasma filaments, which locally induce higher deposition rate, ultimately leading to the growth of sub‐millimeter, hill‐like spots. Surface discharges igniting outwards from filaments were also observed by high‐speed imaging and associated with thinner, ring‐like deposits around the aforementioned spots. These ring‐like areas may have been induced by local consumption of precursor in filaments, leading to a local lack of precursor around the latter and locally preventing film formation from surface discharges. Differences in the surface composition revealed by XPS throughout the different areas of the film were more importantly noted for Si substrates and supported by infrared microscopy. These analyses suggested a heavier fragmentation of the precursor inside filaments, leading to the possible grafting or creation of polar functionalities, such as from carboxylic acid and hydroxyl groups. Overall, this preliminary work demonstrates that atmospheric pressure DBD discharges with organized streamers could be a simple and one‐step route to surface chemical and topographical patterning via polymer deposition and functionalization, ultimately allowing wettability control over sub‐millimeter length scales.

## 4 Experimental Section

### Dielectric Barrier Discharge Setup

**Figure 7:**
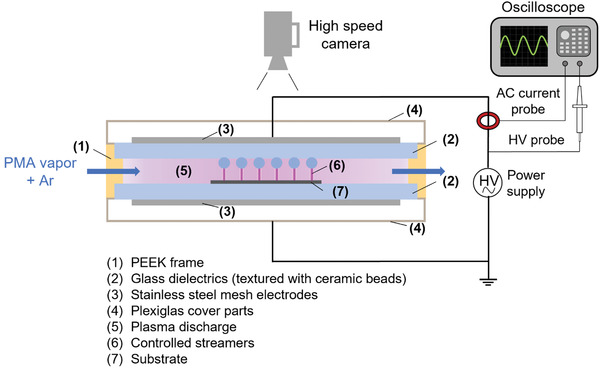
Schematic of the DBD plasma deposition system showing how streamers (6) are immobilized using ceramic beads attached to the upper dielectric insulator.

The atmospheric pressure dielectric barrier discharge (AP‐DBD) device used in this work is shown in **Figure** **7**.

The main structure of the plasma reactor is a polyether ether ketone (PEEK) frame that holds two soda‐lime glass plates (128 × 68 × 3 mm^3^) that act as dielectrics with a spacing of 3 mm. The electrodes are made from stainless‐steel mesh cut into 110 × 55 mm^2^ rectangles and taped on the outer surface of each glass plate. Two Plexiglas blocks placed on the top and bottom of the frame act as seals. As both the mesh electrodes and the Plexiglas are transparent, one can see the plasma discharge by looking from the top of the reactor. The inner face of the upper glass plate was textured with ceramic beads (zirconium oxide/cerium stabilized, 1.4–1.6 mm diameter, 4 mm square pitch) glued at its surface. The liquid precursor (PMA) was placed in a bubbler and heated in an oil bath at 300 K. A mix of Ar and PMA vapor (2 L min^−1^ of primary Ar flow + 0.03 L min^−1^ of Ar diverted through the bubbler) was fed into the 3 mm gap through a lateral gas inlet. For ease of the reader, the argon flow to the PMA bubbler will be referred as *PMA flow* (L min^−1^). The substrate was placed beneath the textured zone of the dielectric for deposition.

### Materials

PMA precursor (98%) was purchased from Alpha Aesar and the plasma gas was 99.99% pure argon (alphagaz 1, Air Liquide). Substrates were silicon wafers and glass coverslips (≈2 × 2 cm^2^) provided by PI‐KEM and Menzel, respectively. Substrates were cleaned with methanol before deposition.

### Discharge Monitoring

*Electrical Measurements*: During experiments, the applied voltage and discharge current were monitored by means of a high‐voltage probe (Tektronix P6015A) and a Rogowski coil (Pearson current monitor model 2877), respectively. Current and voltage curves were recorded for at least one full period of the high voltage with 100 000 points on an oscilloscope (Tektronix DPO 3032). Each set of data is saved in one single file (which contains three columns for time, voltage, and current).

*Statistical Analysis*: For each plasma condition, ten sets of voltage and current curves were recorded. Each file was processed with *MATLAB* to calculate the root‐mean‐square voltage (*V*_rms_), average current (*I*_d_), and average power (*P*) dissipated in the discharge per period of high voltage (T). P is obtained by integration of the instantaneous power over one full cycle of the voltage, multiplied by the frequency (1/T) [see Equation (1)]. Arithmetic mean, standard deviations, and *P*‐values (two‐sided Student test, *n* = 10, *α* = 0.05) were calculated with Excel on the *V*_rms_ and *I*_d_ values obtained from the ten data sets.

(1) $$P=\frac{1}{T}\int_{t_{0}-T/2}^{t_{0}+T/2}V\left(t\right)\cdot I\left(t\right)\mathrm{dt}$$

where *V*(*t*), *I*(*t*), t_0_ and dt are the instantaneous voltage, instantaneous current, center of the cycle, and the time between two acquisition points, respectively.

*High‐Speed Imaging*: A Photron FASTCAM NOVA S6 camera equipped with a Tamron SP AF 90 mm F/2.8 Di Macro 1:1 lens was used to record high‐speed images of the discharge at different speeds (1000, 6400, or 10000 fps). The image overlays presented in Figure 2 were obtained by stacking 500 successive frames using *Sequator* software with the “Trails composition” option.

### Chemical and Physical Characterization of Coatings

*X‐Ray Photoelectron Spectroscopy*: The elemental composition of the samples surface was studied by XPS with 100 µm diameter analysis areas using a PHI – VersaProbe II spectrometer with a monochromatic Al K*α* X‐ray source (1486.6 eV). Pass energies for survey and high‐resolution spectra were 188 eV (1.87 eV step) and 23.5 eV (0.1 eV step), respectively.

*Statistical Analysis*: Each survey and high‐resolution C 1s spectra were cycled four times from 0 to 1200 eV and ten times from 280 to 294 eV, respectively. For all samples, at least three (3–8) surveys and C1s spectra were acquired on each of the “S” and “BS” regions.

Relative surface concentrations were determined using *CasaXPS* software with C 1s and O 1s sensitivity factors of 0.205 and 0.63, respectively.^[^ ^33^ ^]^ Arithmetic means of O 1s/C 1s ratios, standard deviations, and *P*‐values were calculated with *Excel* software. To assess the significant differences between the means obtained for the “S” and “BS” regions, a two‐sided Student test (*n* = 3–8, *α* = 0.05) was used.

The C 1s spectral envelope was fit using *CasaXPS* with Gaussian–Lorentzian peaks, a Shirley background, and four components: C—C/C—H (284.9 ± 0.1 eV), C—O—R/O═C—O—C* (286.6 ± 0.1 eV), C═O/O—C—O (288.0 ± 0.1 eV), COOR (289.0 ± 0.2 eV), with R = alkyl or H). ^[^ ^34^ ^]^ The binding energies were calibrated to 284.9 eV for the hydrocarbon component and the spectra were normalized to the peak maximum. To assess significant differences between the C 1s spectra acquired on the “S” and “BS” areas, their arithmetic means, upper and lower confidence intervals at 95% (CI 95) were plotted in a same graph and compered (see the Supporting Information). At the binding energies where the lower and upper CI 95 do not cross, the means were considered significantly different. The average spectra and CI 95 were calculated with *OriginPro 8.5* software.

*Micro‐Infrared Imaging*: The deposited thin films were also characterized by micro‐infrared imaging (900–3700 cm^−1^) using a Cary 620 FTIR microscope equipped with LN_2_‐cooled HgCdTe detector in reflection mode. The analysis area for IR imaging was 2640 × 2640 µm^2^ with 128 × 128 pixels. IR images at different wavenumbers including both “S” and “BS” areas are shown in the Supporting Information.

*Statistical Analysis*: Infrared images were processed with *MATLAB*. For each spectrum of the image, which is the result of 64 scans, the following operations were executed:

- Baseline at wavenumbers 3690, 3048, 2700, 1898, 1864, 1504, 1320, 992, 904 cm^−1^
- Apodization
- Flatten CO_2_ between 2450 and 2250 cm^−1^
- Scaling to the CH_2_/CH_3_ str. band (3035–2800 cm^−1^)

Spectra with a signal‐to‐noise ratio too low were removed from the image. Specifically, the region around the spot was too thin to generate sufficient IR signals and thus appear in black on the image. In addition, spectra from the craters in the center of the spots, slightly deformed due to scattering effects, are not considered either. For the two regions of interest of the PMA sample (“S” and “BS” areas), rectangles containing ≈100 spectra were selected directly on the image. To assess significant differences between the IR spectra of the two regions, their arithmetic means, upper and lower CI 95 were plotted in one graph and compared. In the wavenumbers range where the lower and upper CI 95 do not cross, the means were considered significantly different. The average spectra and CI 95 were calculated with *OriginPro 8.5* software.

*Water Contact Angles*: WCAs were measured using a Krüss DSA100 goniometer (static mode) and Drop Shape Analysis software. Deionized water droplets were deposited at 400 µL min^−1^ dispensing rate.

*Statistical Analysis*: For nonpatterned PMA coatings (3 µL drop) and on each area of interest (“S,” “AS,” or “BS”) of the patterned PMA samples (0.5 µL drop), five WCA measurements were performed. The arithmetic means and standard deviations of the WCA values were calculated with *Excel* on all five measurements recorded on the investigated area or sample.

*Profilometry*: The topography and thickness of the deposited films were investigated by profilometry using a DektakXT profilometer. Each pass of the stylus over the sample surface was done with a “force” of 0.03 mg and a resolution of 1 µm/point and 2 µm/trace. The spot profile shown in Figure 3b is a trace selected from the spot mapping shown the same figure.

## Conflict of Interest

The authors declare no conflict of interest.

## Supporting information

Supporting Information

## Data Availability

The data that support the findings of this study are available from the corresponding author upon reasonable request.
