# Supplementary material for: Simple and Scalable Chemical Surface Patterning via Direct Deposition from Immobilized Plasma Filaments in a Dielectric Barrier Discharge
Source: Adv Sci (Weinh). 2022 Mar 27;9(15):2200237. doi: 10.1002/advs.202200237 (PMC9130873; doi:10.1002/advs.202200237)
Supplement: Supplementary file 1 — Supporting Information [file ADVS-9-2200237-s001.pdf]

ADVANCED SCIENCE Open Access

Supporting Information

for *Adv. Sci.*, DOI 10.1002/advs.202200237

Simple and Scalable Chemical Surface Patterning via Direct Deposition from Immobilized Plasma Filaments in a Dielectric Barrier Discharge

Annaëlle Demaude, Kitty Baert, David Petitjean, Juliette Zveny, Erik Goormaghtigh, Tom Hauffman, Michael J. Gordon and François Reniers*

Supporting Information

Simple and Scalable Chemical Surface Patterning via Direct Deposition from Immobilized Plasma Filaments in a Dielectric Barrier Discharge.

Annaëlle Demaude, Kitty Baert, David Petitjean, Juliette Sveny, Erik Goormaghtigh, Tom Hauffman, Michael J. Gordon, and François Reniers*

- Guidelines for reproducing deposition with immobilized filaments in other conditions:

The nature of the substrate influences the properties of the filaments. Conductive substrates such as metals with a high secondary emission coefficient will tend to produce higher current and longer lifetime streamers than insulating materials[1], which could damage the substrate surface. It is thus suggested to limit the applied power when using the former type of materials.

The filamentary nature of the discharge depends on the 'plasma gas' used but also on the additional presence and amount of a precursor. For example, Ar is known to lead to filamentary discharges more easily, while He allows obtaining more diffuse discharges[1]. Injecting a precursor generally modifies its filamentary character due to interactions with the plasma gas excited species and the subsequent generation of specific fragments that can influence the microdischarges characeristics[2]. Therefore, depending on the nature of the discharge, the immobilization of the filaments could necessitate an optimization of different plasma parameters (frequency, power, precursor flow) and texturization of the dielectrics (different beads arrangement or different types of texturization, etc.).

For a same mixture of carrier gas and precursor, it is possible to achieve different patterns by varying the spacing between the beads. Examples of coatings deposited at 10 W on Si wafers using a dielectric texturized with beads spaced 2 mm, 4 mm (as in the main paper for

[1] F. Massines, C. Sarra-Bournet, F. Fanelli, N. Naudé, N. Gherardi. *Plasma Process. Polym.* **2012**, *9* (11–12), 1041–1073.
[2] J. Mertens, J. Baneton, A. Ozkan, E. Pospisilova, B. Nysten, A. Delcorte, F. Reniers. *Thin Solid Films* **2019**, 671, 64–76.

comparison) and 8 mm apart are reported in Figure 1a, 1b and 1c, respectively. Pictures of the respective discharges are also presented in Figure 1d to f.

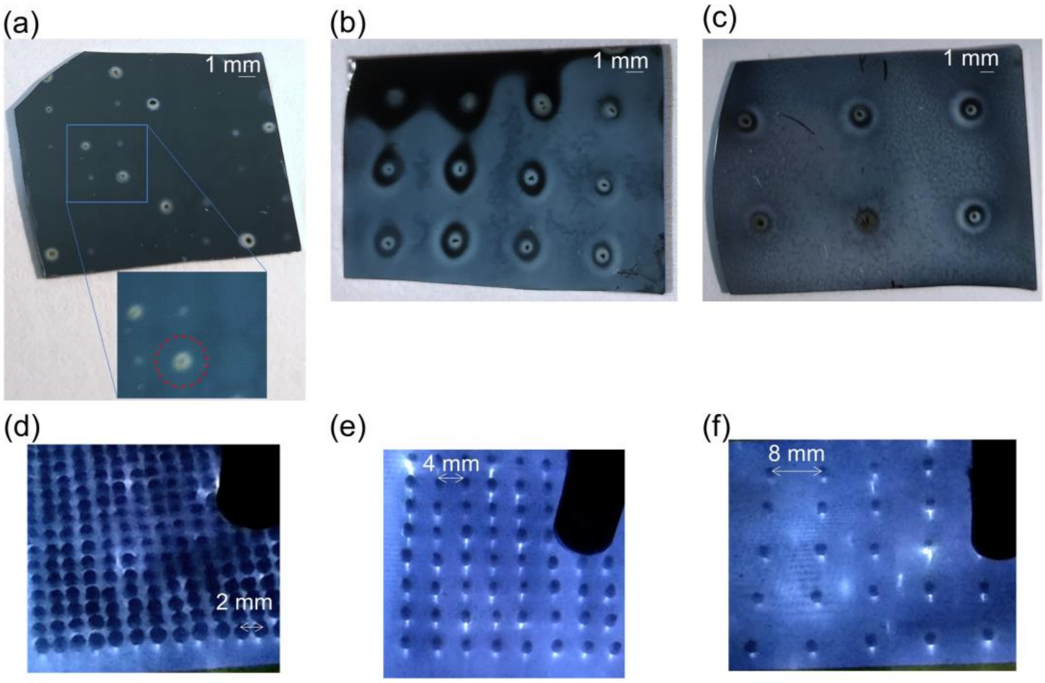

Figure 1. Pictures of PMA plasma deposited films on silicon wafer substrates using an upper dielectric textured with beads displayed in a 2 (a), 4 (b) and 8 (c) mm square pitch. Picture of the corresponding discharges (selected from videos acquired at 60 fps) are presented in (d), (e) and (f). Conditions: Ar flow = 2 L min-1, PMA flow = 0.03 L min-1, f = 24680 Hz, applied power = 10 W, deposition time = 120 s.

In the case where the beads are spaced 8 mm apart (see Figure 1f), filaments are mainly focused under the beads, while few filaments are still also igniting randomly in between the beads. However, a topographical pattern is still obtained. In the case where the beads are spaced 2 mm from each other, the size of the spots is visibly more variable compared to the other samples and the 'AS' and 'BS' regions are less noticeable (see zoomed picture in Figure 1a).

XPS analysis on 'BS' areas of the 2 mm sample showed a very important signal of O and Si compared to the one of C (see Figure 2a), suggesting indeed a very low deposition rate between the beads in this configuration.

Despite the presence of the few free filaments in the 8 mm spaced beads discharge, the chemistry of the resulting film was found different between the 'S' and 'BS' areas. Indeed, as for the S-10W sample discussed in the paper, a significant increase of the C 1s peak signal in

the range of the C–O–R/O=C–O–C* (286.6 ± 0.1 eV, with R= H or alkyl) and C=O/O–C–O (288.0 ± 0.1 eV) binding energies was found in the 'S' area of the 8 mm sample (see Figure 2b).

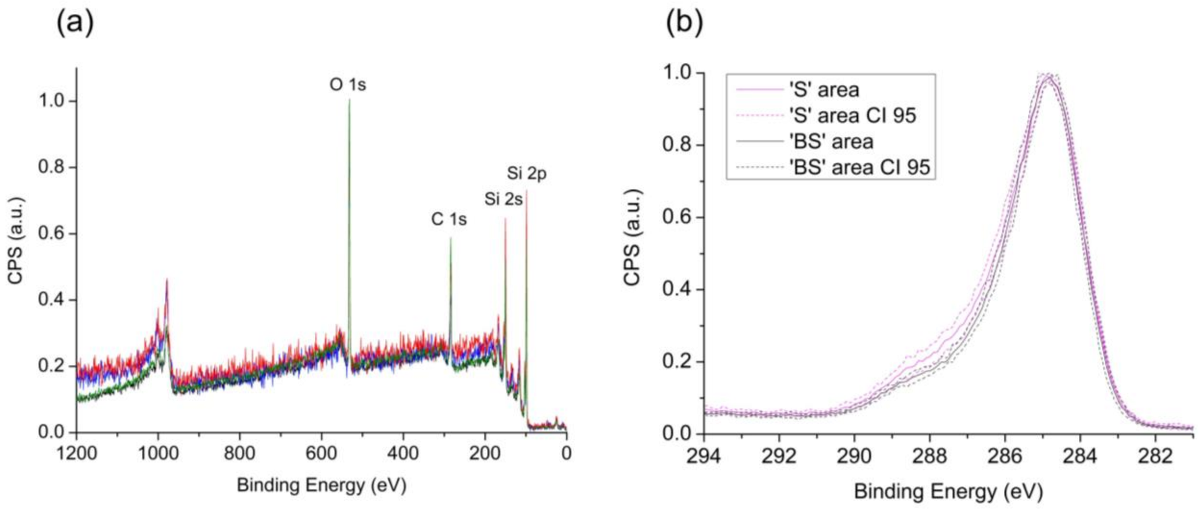

Figure 2. (a) XPS survey spectra (overlay of 5 spectra, normalized to the O 1s peak maximum) acquired between the spots of the PMA coatings deposited with the 2 mm beads pitch. (b) Arithmetic means (solid lines), lower and upper confidence intervals at 95% (dashed lines, n = 5) of high resolution C 1s spectra acquired on the spots ('S' areas) and between the spots ('BS' areas) of the PMA coatings deposited with the 8 mm beads pitch. Conditions: Ar flow = 2 L min-1, PMA flow = 0.03 L min-1, f = 24680 Hz, P = 10 W, deposition time = 120 s.

The XPS analysis were consistent with water contact angles (WCA) measurements on the different areas of the 2 and 8 mm samples. Pictures of the drops during the measurements and mean WCA values are reported in Figure 3. For the 2 mm sample, a hydrophilicity similar to the pristine substrate was observed on the 'BS' areas, although, no precise value of WCA could be obtained because of the distortion of the drop when spreading on the surface. Moreover, the 'S' areas of this sample were too small to precisely deposit a drop and probe their wettability. For the 8 mm sample, as for the S-10 W sample of the paper, hydrophobic and hydrophilic properties were noted on the 'BS' and 'AS+S' areas, respectively.

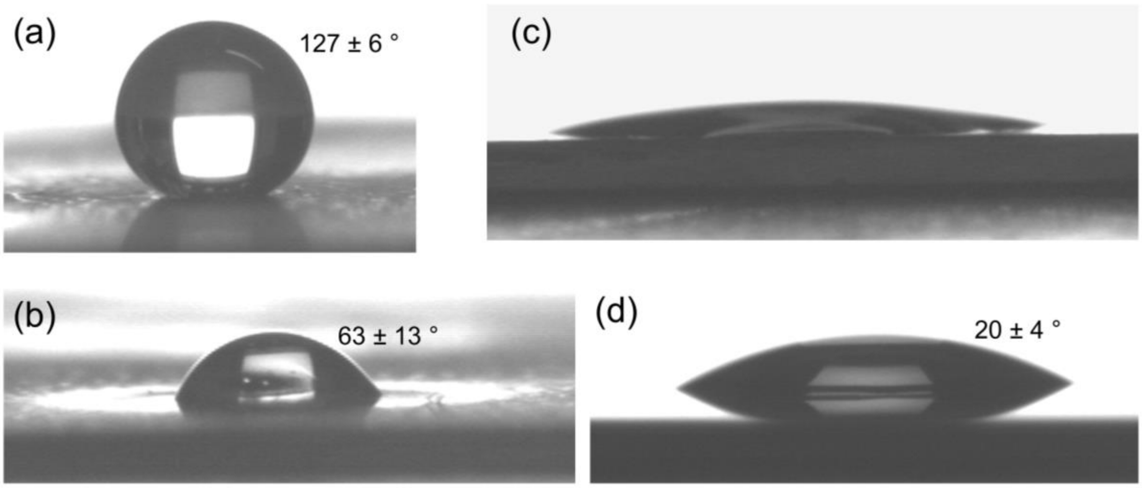

Figure 3. Pictures of a 3 µL water drop deposited between the spots (a) and of a 0.5 µL drop deposited on the spots (‘S’ + ‘AS’ areas) (b) of a PMA coating deposited with the 8 mm beads pitch dielectric. In (c) and (d), picture of a 0.5 µL drop deposited between the spots (‘BS’ area) of a PMA coating deposited with the 2 mm beads pitch dielectric and on a pristine Si substrate, respectively. Numbers refer to the mean WCA measured in the respective areas (arithmetic means ± standard deviations of at least 5 values, respectively). Conditions: Ar flow = 2 L min-1, PMA flow = 0.03 L min-1, f = 24680 Hz, P = 10 W, deposition time = 120 s.

- Profilometry profiles of spots on G-6W and S-10W samples:

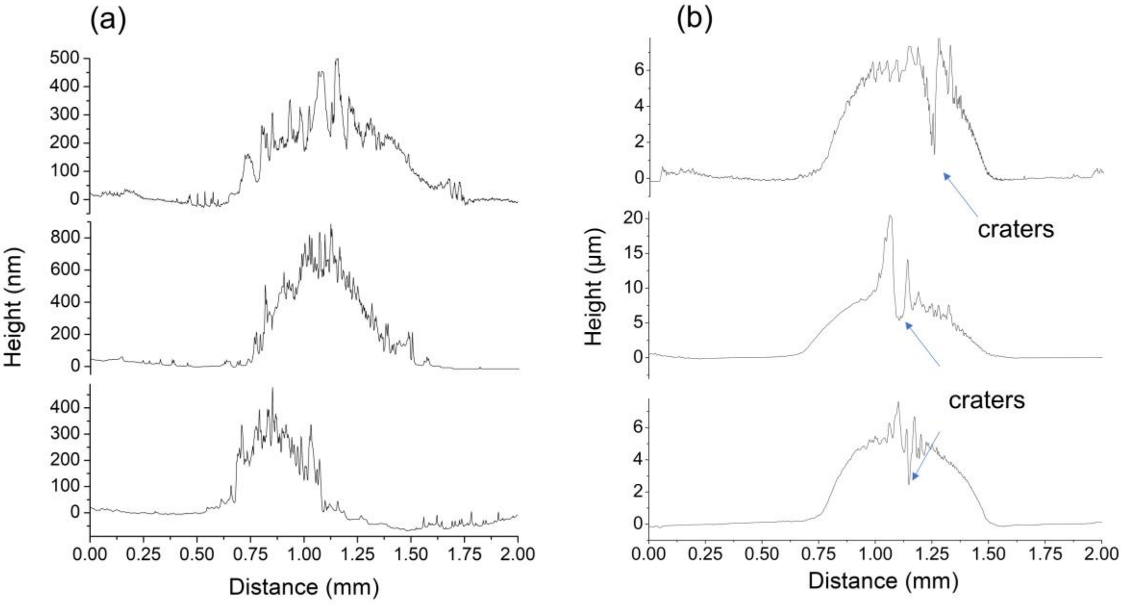

Figure 4. Profilometry profiles of spots from the G-6W (a) and S-10 W (b) coatings discussed in the main paper. Resolution: 0.4 µm/pt. Conditions: Ar flow = 2 L min-1, PMA flow = 0.03 L min-1, f = 24680 Hz, deposition time = 120 s.

The depth of the craters in the center of the S-10W sample could be underestimated as their width is smaller than the diameter of the profilometer stylus, which is thus not able to reach the bottom of the holes. This also seemed to provoke a slight oscillation of the stylus tip near the craters and thus a local overestimation of the thickness.

- Current and Voltage curves

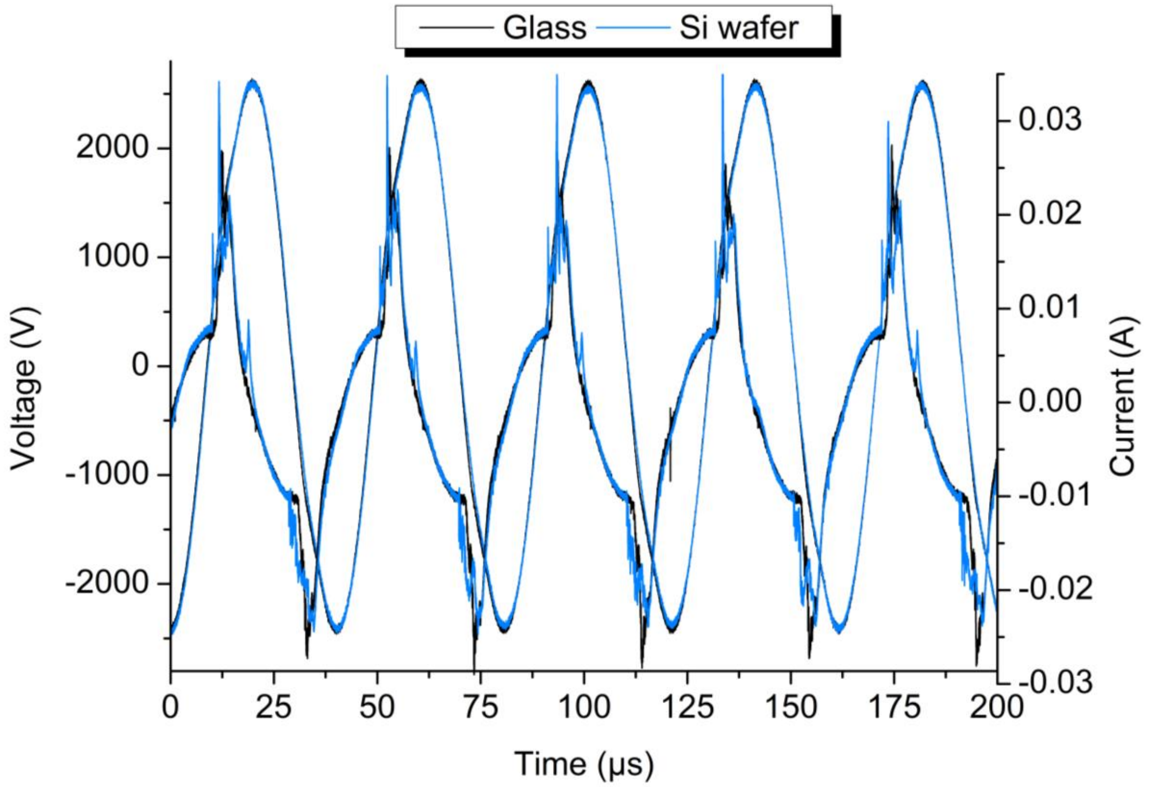

Figure 5. Current (sinusoidal + peaks curve) and voltage (“simple” sinusoidal curve) recorded during deposition with a Si wafer or a glass substrate. Conditions: Ar flow = 2 L min-1, PMA flow = 0.03 L min-1, f = 24680 Hz, P = 6W.

- Supplementary IR and XPS data:

X-ray photoelectron spectroscopy:

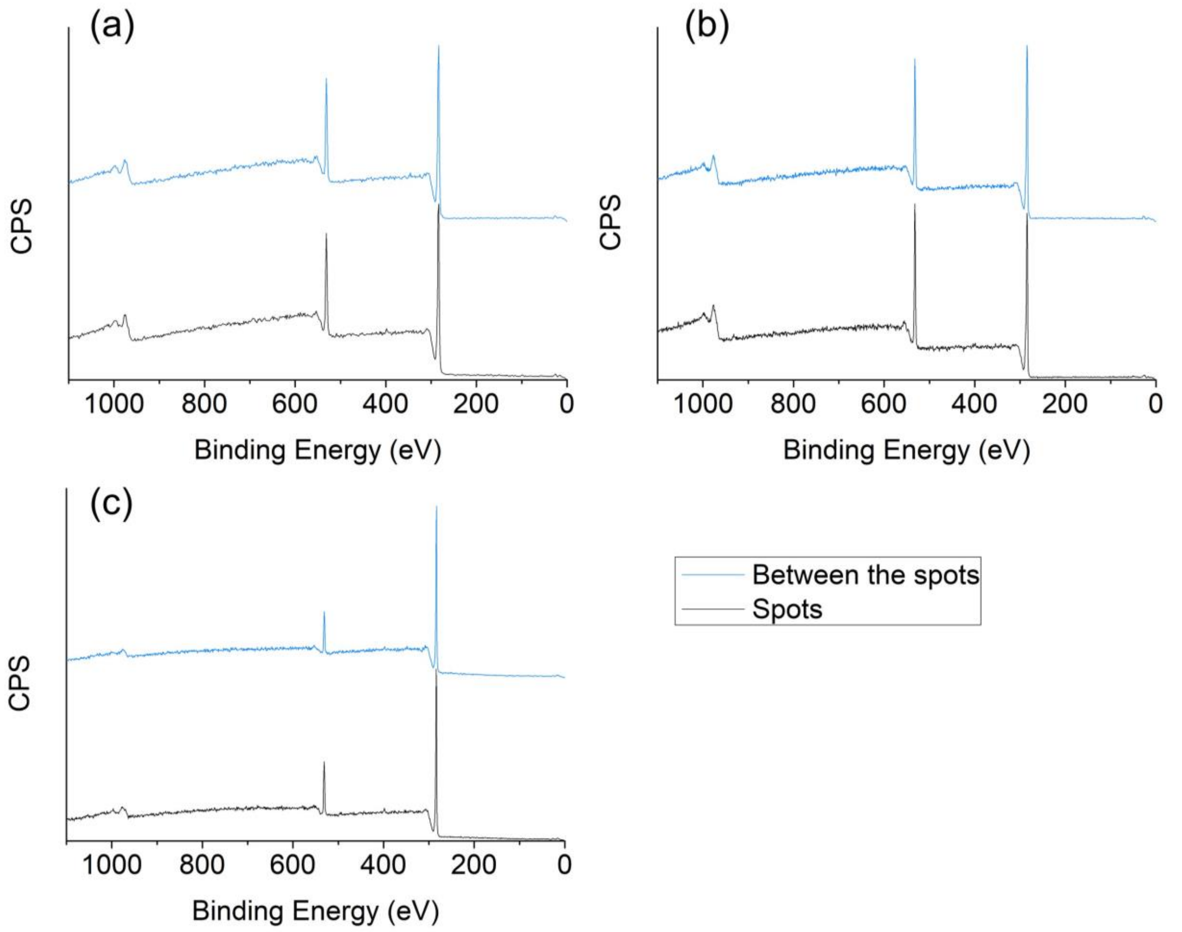

Figure 6. XPS survey spectra acquired on the spots and between the spots on PMA coatings deposited at 10W (a) or 6 W (b) on Si substrate and 6W on glass (c) substrates. Conditions: Ar flow = 2 L min-1, PMA flow = 0.03 L min-1, f = 24680 Hz, deposition time = 120 s.

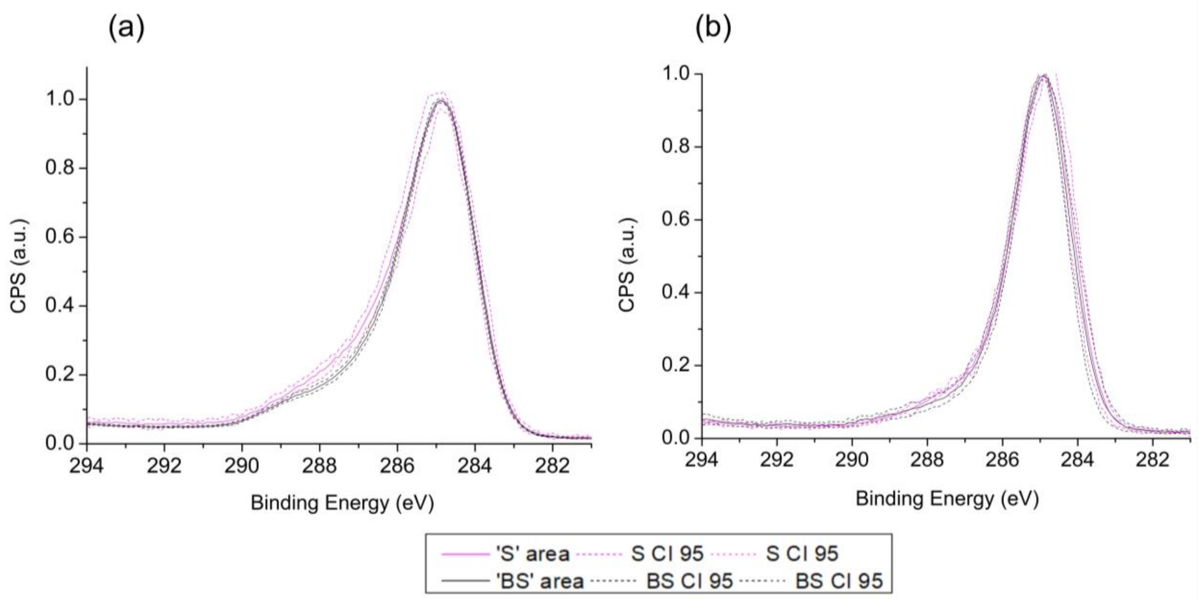

Figure 7. Arithmetic means (solid lines), lower and upper confidence intervals at 95% (dashed lines, n = 3-8) of high resolution C 1s spectra acquired on the spots and between the spots of PMA coatings deposited at 10 W on Si (a) and 6 W on glass (b). Conditions: Ar flow = 2 L min-1, PMA flow = 0.03 L min-1, f = 24680 Hz, deposition time = 120 s.

Infrared imaging:

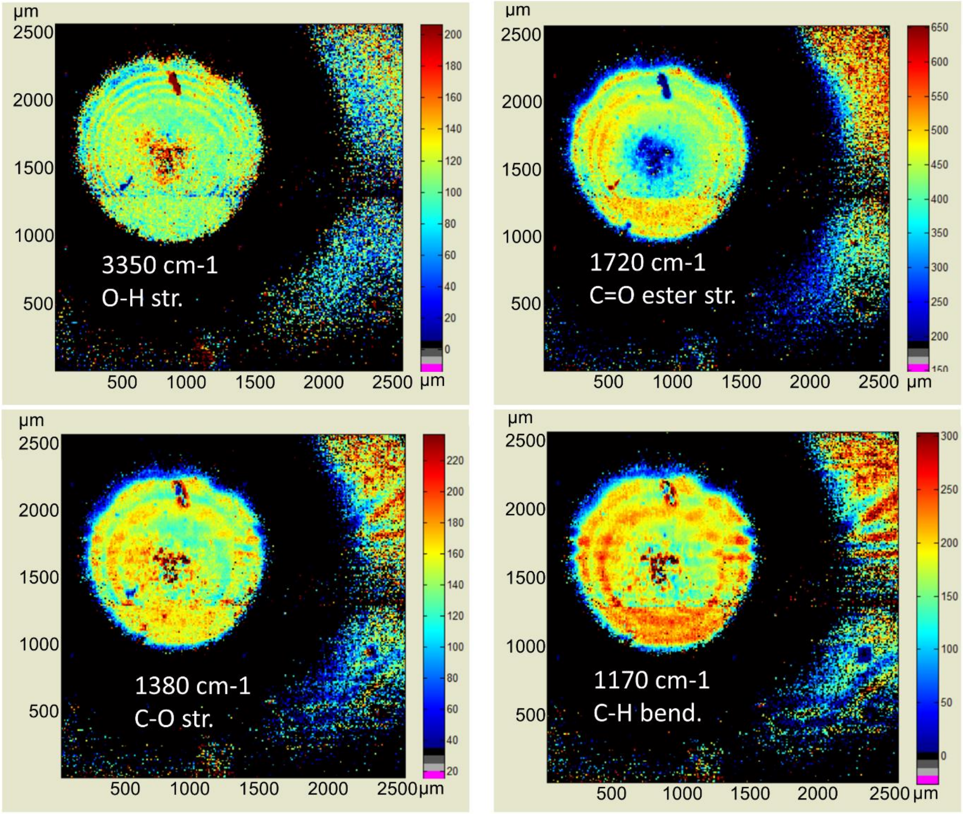

Figure 8. IR images at 3350, 1720, 1380 and 1170 cm^{-1} of a patterned PMA coating deposited at 10 W on aluminum showing a region containing a spot. Conditions: Ar flow = 2 L min-1, PMA flow = 0.03 L min-1, f = 24680 Hz, deposition time = 120 s.
